# Relationship between Meteorin-like protein and type 2 diabetes mellitus: an update and meta-analysis

**DOI:** 10.1530/EC-25-0452

**Published:** 2025-09-26

**Authors:** Jing Xie, Yanhua Jiang, Jiayi Yao, Bin Feng, Xin Sun

**Affiliations:** ^1^ Department of Endocrinology, First Affiliated Hospital of Soochow University, Suzhou, P.R. China; ^2^ Department of Ophthalmology, Fourth People’s Hospital of Shenyang, Shenyang, P.R. China

**Keywords:** Meteorin-like, type 2 diabetes mellitus, Metrnl, Subfatin

## Abstract

**Background:**

Meteorin-like protein (Metrnl) is considered a novel adipokine, which plays an important role in the occurrence and development of type 2 diabetes mellitus (T2DM). In a previous meta-analysis, no significant difference in circulating Metrnl levels was found between T2DM patients and normal glucose tolerance individuals. However, these meta-analyses included limited studies, and more studies about Metrnl and T2DM were published recently. Therefore, the association between Metrnl and T2DM remains uncertain.

**Aim:**

This study aimed to systematically and comprehensively update the relationship between circulating Metrnl levels and T2DM.

**Methods:**

A systematic search was conducted on the Web of Science, Wiley Online Library, PubMed, and Ovid databases using the terms ‘type 2 diabetes mellitus’ in combination with ‘Metrnl’. Meta-analysis results were reported as standardized mean differences (SMDs) with corresponding 95% confidence intervals (CIs).

**Results:**

In total, 17 studies, including 649 patients with T2DM and 396 healthy controls, were included in the meta-analysis. We found that circulating Metrnl levels were lower in patients with T2DM than in healthy individuals (SMD: −0.47, 95% CI: (−0.98, 0.05); prediction interval: (−3.66, 2.73)). Moreover, serum Metrnl levels in patients with T2DM were significantly lower than in healthy individuals (SMD: −0.91, 95% CI: (−1.60, −0.22); prediction interval: (−5.03, 3.21)).

**Conclusions:**

This study aimed to systematically and comprehensively update the relationship between circulating Metrnl levels and T2DM. A clearer understanding of the role of Metrnl in T2DM could facilitate the development of effective treatments.

## Introduction

In recent years, the global prevalence of diabetes mellitus has been rising steadily. In 2021, an estimated 521 million people worldwide had diabetes mellitus, including approximately 485 million individuals aged 20–79 years. The number of people with diabetes mellitus is expected to reach 1.31 billion by 2050 (1). Chronic hyperglycemia can lead to complications affecting the eye, nerves, kidneys, and cardiovascular system, posing a serious threat to human health. Insulin resistance is closely associated with type 2 diabetes mellitus (T2DM) (2). Adipose tissue not only can store fat, but also is an endocrine organ that can secrete a variety of bioactive factors, which are collectively referred to as adipokines. They play an important role in regulating glucose and lipid metabolism, inflammation, immunity, cardiovascular function, and cancer (3). Meteorin-like protein (Metrnl) is considered a novel adipokine, which plays an important role in the occurrence and development of diabetes mellitus by affecting glucose metabolism and insulin resistance, thereby affecting glucose and lipid metabolism and energy homeostasis in the body (4).

Recent studies have shown that Metrnl is closely related to the occurrence and development of diabetes mellitus. Some researchers found that Metrnl levels were significantly lower in patients with diabetes mellitus than in healthy controls, whereas other researchers found opposite results (5, 6, 7). A meta-analysis included nine studies with 867 T2DM patients and 831 normal glucose tolerance controls. In this meta-analysis, no significant difference in circulating Metrnl levels was found between T2DM patients and normal glucose tolerance individuals (8). Another meta-analysis showed that there was no significant association between serum Metrnl levels and risk of T2DM in patients compared with healthy controls (9). However, these meta-analyses included limited studies, and more studies about Metrnl and T2DM were published recently. Therefore, the association between Metrnl and T2DM remains uncertain. This study aimed to systematically and comprehensively update the relationship between circulating Metrnl levels and T2DM.

## Methods

### Literature search

A literature search was conducted using the Web of Science, Wiley Online Library, PubMed, and Ovid databases. The search range covered studies exploring the connection between Metrnl and T2DM, published up to March 2025. The terms ‘Meteorin-like’, ‘Metrnl’, ‘Subfatin’, ‘Cometin’, and ‘diabetes mellitus’ were used for the title or abstract search. Eligible studies were limited to those published in English. The references of the included studies were traced to supplement the relevant literature. The present study was registered in the PROSPERO database (CRD420251004447) in accordance with the Cochrane Handbook for Systematic Reviews of Interventions. All necessary items reported for systematic reviews and meta-analyses are presented in the Supplementary Data (see section on Supplementary materials given at the end of the article).

### Inclusion criteria

Meta-analysis was performed on studies that met the following criteria: availability of data on circulating Metrnl levels in patients with T2DM and healthy controls, case–control or cohort design, and publication language limited to English.

### Exclusion criteria

Inaccessible or duplicate publications, those containing incomplete or non-convertible data, or studies containing intervention measures in the experimental or control groups not meeting the study requirements were excluded. In addition, studies with major design flaws, as well as animal studies, reviews, meeting summaries, case reports, and editorials were excluded.

### Data extraction and risk of bias

Two researchers independently screened the literature and extracted and cross-checked the data. Disagreements were resolved through discussion or consultation with a third researcher. The literature screening was performed by reading the titles. After eliminating the irrelevant literature, the abstracts and full texts were further read to determine the inclusion criteria. If necessary, the original study authors were contacted via mail or telephone to obtain missing but relevant information. Extracted data included key study details such as the research title, first author, publication year, location, sample size, age of each group, and indicators.

The Newcastle–Ottawa scale (NOS) was independently used by two researchers to assess the risk of bias in the included studies, and the outcomes were cross-validated (10). In the event of disagreement, a third researcher assisted in the judgment. The NOS encompasses three dimensions and eight items, with a total score of 9. The higher the score, the lower the risk of bias. A score ranging from 7 to 9 was regarded as high quality, 4–6 as moderate quality, and ≤3 as low quality.

### Statistical analysis

The results are presented as the standardized mean difference (SMD) and the corresponding 95% confidence interval (CI). Heterogeneity was assessed based on *I*^2^ and *P* values. In cases where there was no statistical heterogeneity among the outcomes of each study, a fixed-effects model was adopted for the meta-analysis. If statistical heterogeneity existed among the results of each study, the source of the heterogeneity was further explored. After eliminating the impact of obvious clinical heterogeneity, a random-effects model was used for the meta-analysis. Heterogeneity was assessed using subgroup or sensitivity analyses. Begg’s and Egger’s tests were used to identify publication biases in the included studies. Differences were considered statistically significant at *P* < 0.05. Analyses were performed using Stata Release 12.0 (StataCorp LLC, USA).

## Results

### Literature search and study selection

**Figure 1:**
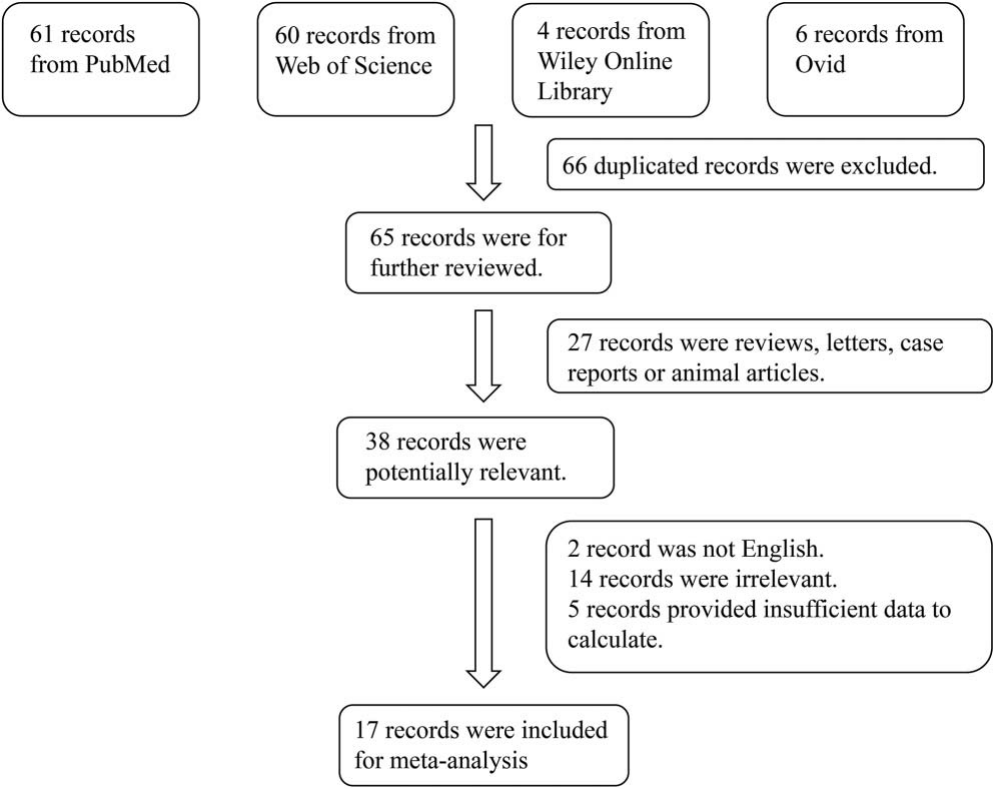
Flowchart of the detailed procedure for the inclusion or exclusion of selected studies.

A total of 131 relevant studies were retrieved from the Web of Science, Wiley Online Library, PubMed, and Ovid databases. Among these, 66 duplicates were removed, and 27 were excluded after screening titles and abstracts. In addition, 38 studies were excluded after full-text review. Consequently, 17 studies, including 649 patients with T2DM and 396 controls, were included in the meta-analysis (5, 6, 7, 11, 12, 13, 14, 15, 16, 17, 18, 19, 20, 21, 22, 23, 24, 25). A flow diagram illustrating the study selection process is shown in Fig. 1. The characteristics of the included studies are summarized in Table 1. All 17 studies included in this meta-analysis fulfilled NOS criteria.

**Table 1:** Characteristics of the published studies included in the meta-analysis.

Author	Publication year	Region	Number (*n*)		Meteorin-like protein level		Sex (M/F)		Age (years)		Sample
			Case	Control	Case	Control	Case	Control	Case	Control	
Lee JH	2018	Korea	46	47	59 ± 23	108 ± 27	21/25	18/29	37.3 ± 4.6	49.0 ± 15	Serum
Dadmanesh M	2018	Iran	63	41	73.89 ± 33.6	95.33 ± 33.56	41/22	29/12	58.6 ± 7.8	56.4 ± 7.5	Serum
Fadaei R	2018	Iran	52	50	66.67 ± 34.59	80.7 ± 23.53	34/18	39/11	58.6 ± 1.1	57.6 ± 1.2	Serum
Zheng SL	2018	China	9	11	347.3 ± 55.8	432.8 ± 76.02	5/4	6/5	50.0 ± 11.0	34.6 ± 2.0	Serum
Chung HS	2018	Korea	400	400	1,260.73 ± 83.16	1,146.73 ± 238.45	213/187	154/246	59.0 ± 10.1	58.3 ± 10.4	Plasma
AlKhairi I	2019	Kuwait	104	123	1,263.52 ± 254.65	1,198.58 ± 269.28	55/49	45/78	52.3 ± 0.9	41.8 ± 1.1	Plasma
C Wang	2019	China	59	30	213.57 ± 165.63	145.14 ± 70.83	34/25	18/12	55.9 ± 9.6	56.0 ± 4.1	Serum
K Wang	2019	China	40	40	279.83 ± 146.53	142.35 ± 69.59	24/16	22/18	52.1 ± 7.9	50.7 ± 5.4	Serum
El-Ashmawy HM	2019	Egypt	94	89	61.17 ± 18.16	112.42 ± 31.71	40/54	47/42	51.6 ± 1.7	52.1 ± 1.61	Serum
Onalan E	2020	Turkey	100	50	4.21 ± 10.55	21.16 ± 17.09	38/62	25/25	53.9 ± 11.18	49.94 ± 12.51	Serum
Wang R	2020	China	221	74	116 ± 9.75	138 ± 15.53	112/109	42/32	58.18 ± 11.87	58.34 ± 7.86	Serum
Timurkaan M	2022	Turkey	60	60	2.02 ± 0.9	2.65 ± 1.22	30/30	29/31	50.3 ± 5.76	47.6 ± 7.64	Serum
Tuncer KK	2022	Turkey	30	30	4.41 ± 3.25	2.19 ± 0.85	-	-	54.63 ± 10.8	42.1 ± 8.8	Plasma
Gungor Kobat S	2023	Turkey	40	20	3 ± 0.95	2.29 ± 0.42	16/24	9/11	66.51 ± 8.62	66.55 ± 10.30	Plasma
Khajebishak Y	2023	Iran	32	31	82.5 ± 16.37	144.4 ± 18.52	7/25	8/23	51.9 ± 10.8	42.1 ± 11.1	Serum
Phuong L	2024	Vietnam	86	71	757 ± 107.5	706 ± 109	32/54	49/22	55.64 ± 11.42	54.39 ± 8.34	Plasma
Yao C	2024	China	80	60	574 ± 365.74	322 ± 192	52/28	32/28	53.23 ± 14.38	52.30 ± 10.13	Serum

### Results of meta-analysis

**Figure 2:**
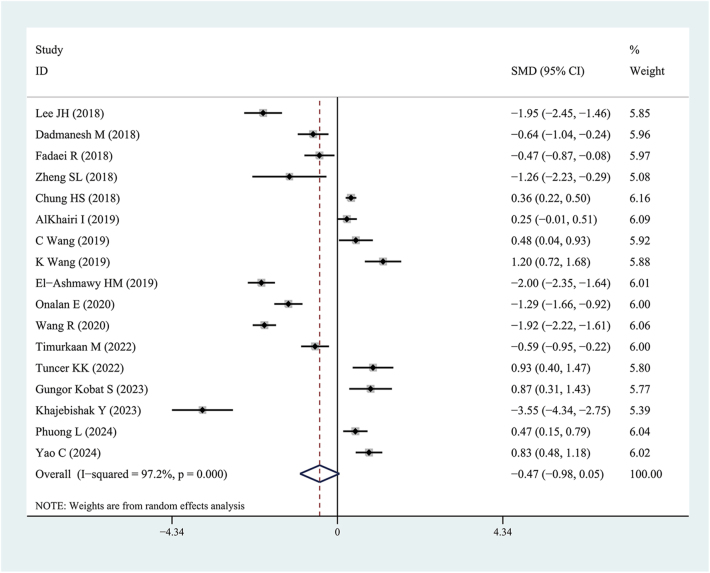
Forest plots of circulating Meteorin-like protein levels in patients with type 2 diabetes mellitus compared to healthy individuals. The diamond represents the pooled SMDs at 95% CI. SMD, standardized mean difference; CI, confidence interval.

Circulating Metrnl levels in patients with T2DM were lower than in healthy individuals (SMD: −0.47, 95% CI: (−0.98, 0.05); prediction interval: (−3.66, 2.73); *I*^2^ = 97.2%). The forest plots of the results are shown in Fig. 2.

### Meta-regression analysis

Because of high heterogeneity in our meta-analysis, we performed the meta-regression analysis according to publication year, sample size, sample type, and reagent. We found the sample type might be an important influencing factor for the observed heterogeneity in this meta-analysis (0.17, 2.79; *P* = 0.03) (Table 2).

**Table 2:** Meta-regression analysis results.

Covariate	*β*	SE	95% CI	*P*
Publication year	0.10	0.15	−0.22, 0.42	0.51
Sample size	0.00	0.00	0.00, 0.00	0.72
Sample type	1.48	0.62	0.17, 2.79	0.03
Duration of disease	0.04	0.08	−0.14, 0.21	0.66
Reagent	0.16	0.17	−0.21, 0.54	0.36
Region	0.29	0.17	−0.08, 0.66	0.12
Body mass index	−0.14	0.13	−0.42, 0.13	0.27

### Subgroup analysis

**Figure 3:**
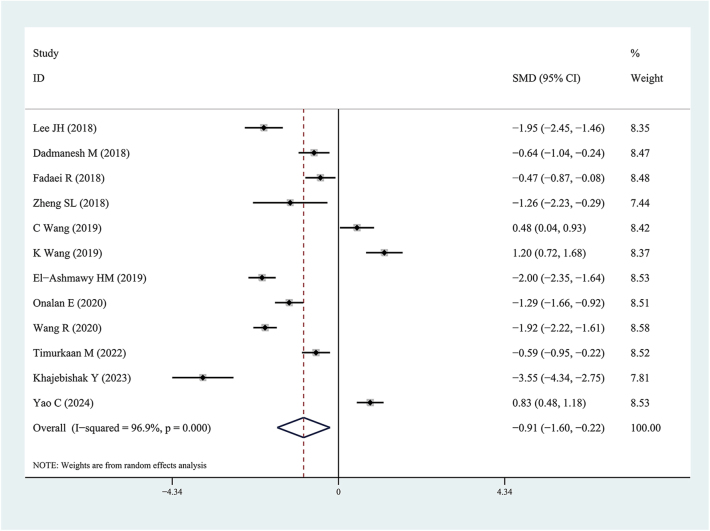
Forest plots of serum Meteorin-like protein levels in patients with type 2 diabetes mellitus compared to healthy individuals. The diamond represents the pooled SMDs at 95% CI. SMD, standardized mean difference; CI, confidence interval.

**Figure 4:**
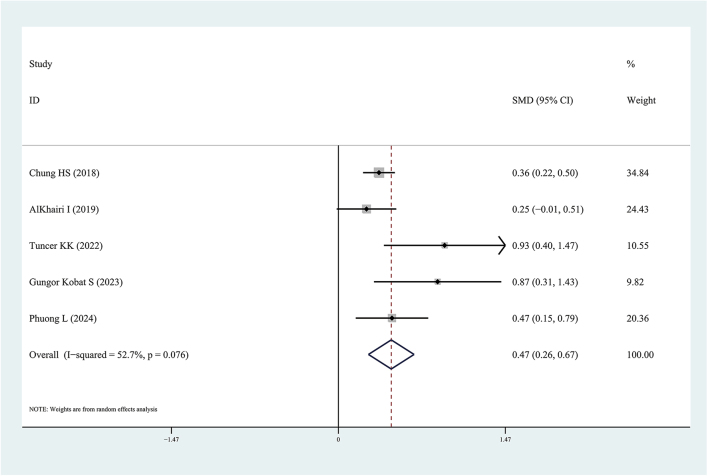
Forest plots of plasma Meteorin-like protein levels in patients with type 2 diabetes mellitus compared to healthy individuals. The diamond represents the pooled SMDs at 95% CI. SMD, standardized mean difference; CI, confidence interval.

We performed subgroup analysis according to the results of the meta-regression analysis. In subgroup analysis, we found serum Metrnl levels in patients with T2DM were significantly lower than in healthy individuals (SMD: −0.91, 95% CI: (−1.60, −0.22); prediction interval: (−5.03, 3.21); *I*^2^ = 96.9%) (Fig. 3). Therefore, we found plasma Metrnl levels in patients with T2DM were significantly higher than in healthy individuals (SMD: 0.47, 95% CI: (0.27, 0.67); prediction interval: (−0.93, 1.86); *I*^2^ = 52.7%) (Fig. 4).

### Publication bias

A sensitivity analysis was conducted by excluding individual studies. The results showed that the combined effect size remained stable, indicating the robustness of the findings (Supplementary Figs S1, S2, S3). A thorough and comprehensive database search was performed. Tests for the presence of publication bias in the results of systematic reviews were performed. Egger’s and Begg’s tests were used to assess publication bias, and no significance was detected (*P* = 0.17 and 0.65, respectively).

## Discussion

This study aimed to systematically and comprehensively update the relationship between circulating Metrnl levels and T2DM. In a previous meta-analysis, no significant difference in circulating Metrnl levels was found between T2DM patients and normal glucose tolerance individuals (8). A total of 17 independent studies were included in our meta-analysis. We found that circulating Metrnl levels were lower in patients with T2DM than in healthy individuals. Moreover, serum Metrnl levels in patients with T2DM were significantly lower than in healthy individuals.

Metrnl, also known as Subfatin and Cometin, was first identified as a neurotrophic factor belonging to the nickel striatin family of neurotrophic regulators. It is located in the region of mouse chromosome 11qE2 and human chromosome 17q25.3 (25). Metrnl is expressed in various tissues and organs, including adipose tissue, skeletal muscle, mucosal tissue, liver, heart, spleen, and central nervous system (26). The physiological functions of Metrnl are complex and diverse. It was initially reported to possess neurotrophic activity and regulate the development and function of the nervous system (25), and subsequent studies have shown that Metrnl also plays a crucial role in immunity and metabolism (27). In addition, it can improve adipose tissue function, combat obesity-induced insulin resistance, and promote energy expenditure, thereby improving glucose metabolism (28).

Metrnl can promote glucose uptake. Lee *et al.* showed that the intraperitoneal injection of Metrnl improved abnormal glucose tolerance in obese and diabetic mice (29). Metrnl increased glucose transporter 4 expression in an AMP-activated protein kinase a2 (AMPKa2)-dependent manner. Phosphorylated histone deacetylase 5 (HDAC5), a transcriptional repressor of GLUT4, interacts with the 14-3-3 protein, resulting in the sequestration of HDAC5 in the cytoplasm and eliminating the inhibitory effect of HDAC5 on GLUT4. It activates GLUT4 transcription, promotes glucose uptake by cells, and eventually decreases blood glucose levels. Metrnl increased insulin sensitivity. Metrnl knockout in pancreatic β cells leads to insulin resistance. Jung *et al.* showed that Metrnl improves palmitic acid-induced inflammation and insulin resistance in C2C12 myotubes and skeletal muscle cells of high-fat diet (HFD)-fed mice. Metrnl ameliorated lipid-induced inflammation and insulin resistance through AMPK- or peroxisome proliferator-activated receptor δ (PPARδ)-dependent signaling in mouse skeletal muscle (30). Li *et al.* constructed adipocyte-specific Metrnl overexpression and knockout mouse models and observed that Metrnl knockout aggravated HFD-induced insulin resistance, whereas Metrnl overexpression prevented HFD-induced insulin resistance or leptin deficiency. PPARγ inhibitor and PPARγ knockdown abolished the improvement of insulin resistance by Metrnl, suggesting that the increase in insulin sensitivity by Metrnl is mediated by PPARγ signaling (28).

In previous meta-analyses, no significant difference in circulating Metrnl levels was found between T2DM patients and healthy individuals (8, 9). In our meta-analysis, meta-regression analysis revealed that sample type may serve as a critical factor contributing to the observed heterogeneity. Consequently, we conducted a subgroup analysis stratified by sample type to further explore this association. In the subgroup analysis, we found that serum Metrnl levels in patients with T2DM were significantly lower, while plasma Metrnl levels in patients with T2DM were significantly higher than in healthy individuals. The results showed opposing directions of association for serum and plasma samples. This is an important finding of our meta-analyses. These previous meta-analysis included studies with different sample types, which might have affected the results. We sought to investigate the underlying reasons for this observation. This difference may stem from various aspects such as sample processing, physiological and pathological factors, and measurement methods. In the future, *in vitro* experiments could be conducted to examine the effects of anticoagulants or the unique serum/plasma environment of T2DM patients on the recovery rate or stability of Metrnl detection.

Metrnl is associated with insulin resistance and T2DM. It also plays an important role in the pathogenesis and prognosis of diabetes mellitus. It affects the metabolism and function of various tissues and organs and plays an important role in maintaining the homeostasis of insulin secretion and energy metabolism. In the future, it is expected to be a therapeutic target for T2DM and provide a new approach for its diagnosis and treatment. However, the relationship between Metrnl and T2DM remains unclear, as does the exact mechanism of its effects; the regulation of insulin resistance and T2DM is still unclear. Further studies and clinical trials are required to verify the feasibility and effectiveness of Metrnl as a potential target for treating T2DM and insulin resistance. In-depth exploration of the relationship between Metrnl and insulin resistance, as well as the occurrence and development of T2DM, is crucial for understanding the molecular mechanisms that regulate insulin synthesis and energy metabolism homeostasis. These results are expected to provide valuable insights for the development of novel T2DM treatment strategies.

The present study had some limitations. First, the duration of T2DM differed among the studies. Second, BMI- or age-matched healthy controls were not used in some studies. Third, most of the included studies were conducted in China and Turkey, leading to a scarcity of data from other populations. Future research should actively break down geographical barriers through global collaboration, data sharing, and refined statistical analysis, ultimately unveiling the complete picture of Metrnl’s role across all human populations. However, owing to the restricted inclusion in the study, a firm conclusion could not be reached. It is crucial to interpret the results of this meta-analysis with caution, as all these factors could have influenced them; thus, further RCT research is needed.

## Conclusion

This study aimed to systematically and comprehensively update the relationship between circulating Metrnl levels and T2DM. Full characterization of the role of Metrnl in T2DM will significantly contribute to improved treatment strategies.

## Supplementary materials





## Declaration of interest

The authors declare that there is no conflict of interest that could be perceived as prejudicing the impartiality of the work reported.

## Funding

This work did not receive any specific grant from any funding agency in the public, commercial, or not-for-profit sectors.

## Data availability

The original contributions presented in the study are included in the article or Supplementary materials. Further inquiries can be directed to the corresponding authors.
