# Supplementary material for: Relationship between Meteorin-like protein and type 2 diabetes mellitus: an update and meta-analysis
Source: Endocr Connect. 2025 Sep 26;14(9):e250452. doi: 10.1530/EC-25-0452 (PMC12478296; doi:10.1530/EC-25-0452)
Supplement: Supplementary file 2 [file supplementary_figures.pdf]

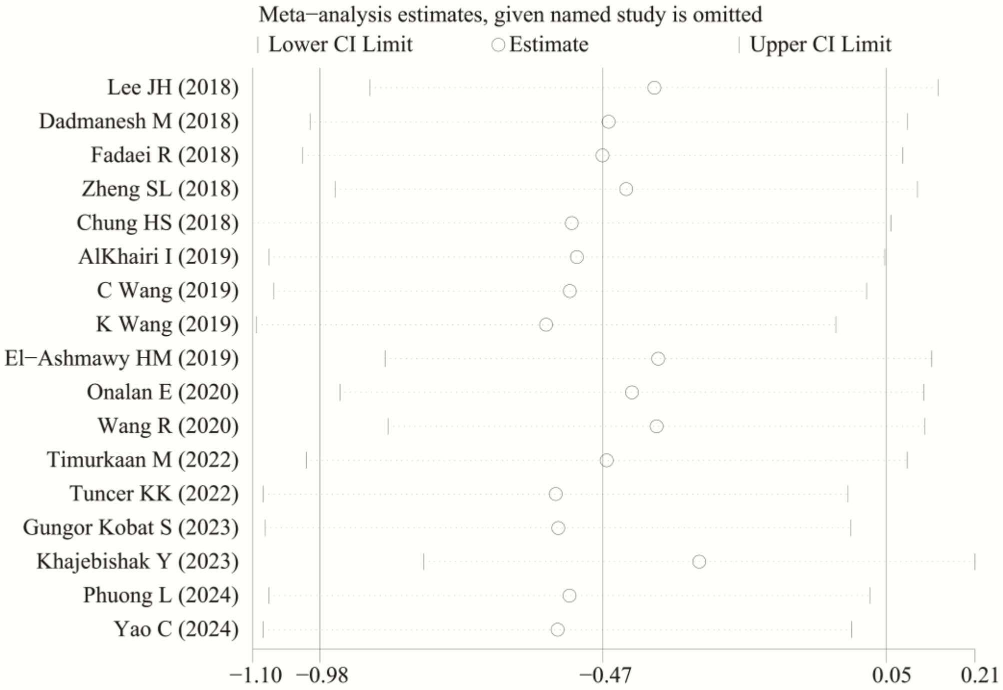

Supplement Figure S1. The sensitivity analysis results of circulating Meteorin-like level in patients with Type 2 diabetes mellitus compared to healthy individuals

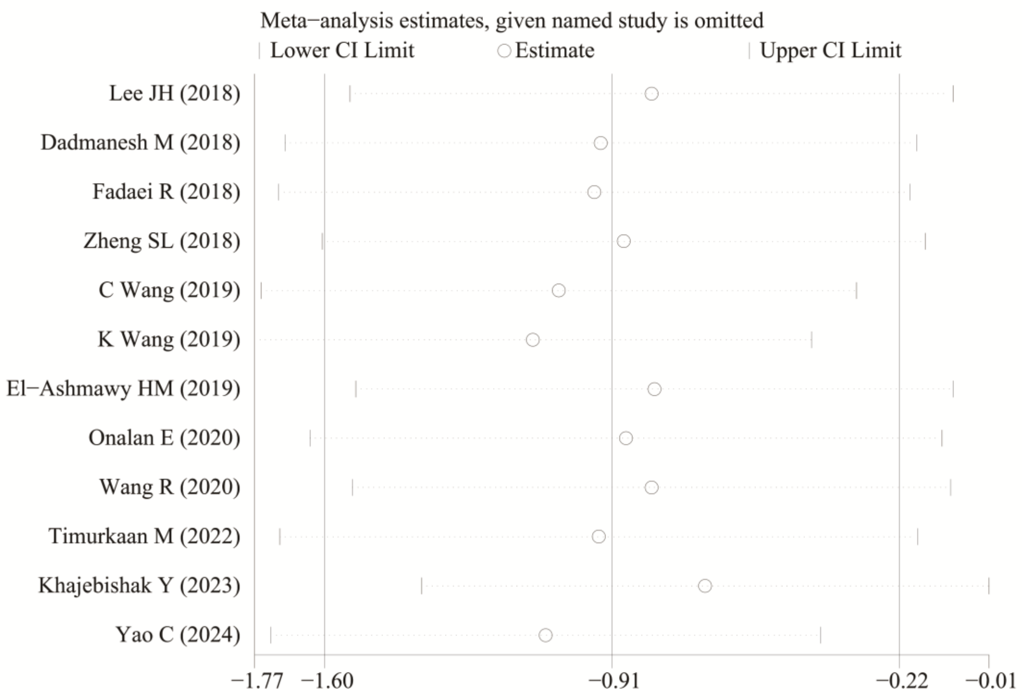

Supplement Figure S2. The sensitivity analysis results of serum Meteorin-like level in patients with Type 2 diabetes mellitus compared to healthy individuals

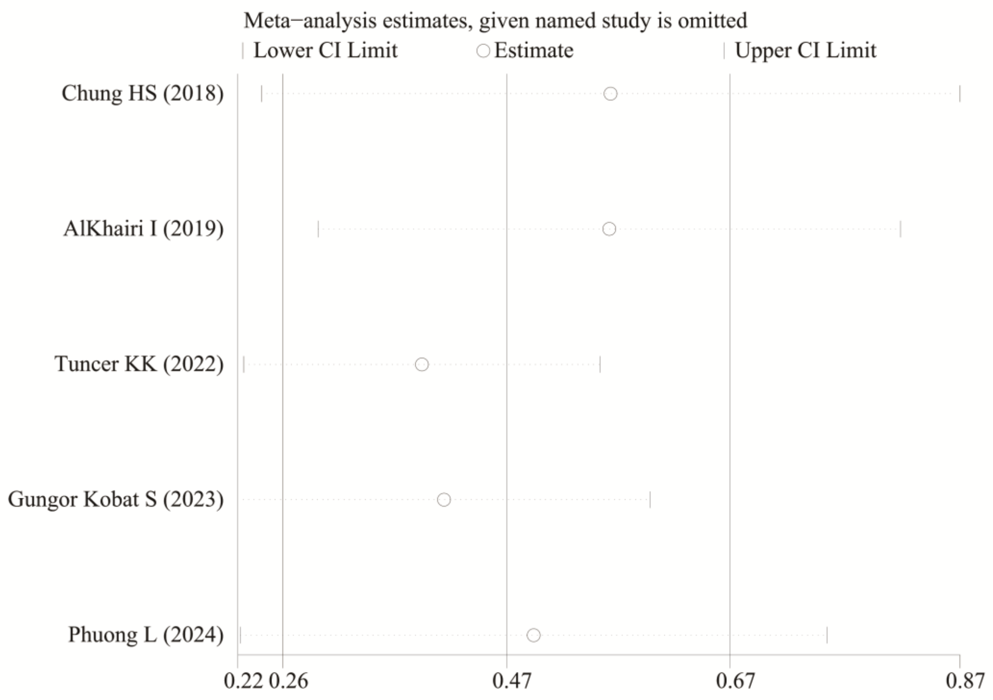

Supplement Figure S3. The sensitivity analysis results of plasma Meteorin-like level in patients with Type 2 diabetes mellitus compared to healthy individuals
